# A Direct Current-to-Digital Converter IC for Luminescence-Based Detection Toward an Energy Efficient Transcutaneous Carbon Dioxide Sensor Wearable

**DOI:** 10.1109/ojsscs.2025.3646593

**Published:** 2025-12-19

**Authors:** TUNA B. TUFAN, BENJAMIN LARKIN, JOHN McNEILL, ULKUHAN GULER

**Affiliations:** Department of Electrical and Computer Engineering, Worcester Polytechnic Institute, Worcester, MA 01609, USA

**Keywords:** Direct current-to-digital converter, dual lifetime referencing, luminescence sensing, transcutaneous sensing, wearable

## Abstract

Continuous monitoring of arterial carbon dioxide is critical for assessing respiratory function and detecting ventilation inefficiencies. Arterial blood gas analysis, the clinical gold standard, is invasive and limited to intermittent measurements in hospital settings. Transcutaneous carbon dioxide sensing offers a noninvasive alternative by measuring carbon dioxide diffusing through the skin, which strongly correlates with arterial carbon dioxide. However, conventional transcutaneous sensors require bulky bedside monitors and heating elements, making them unsuitable for wearable applications. This work presents the first integrated circuit implementation of a ratiometric time-domain dual lifetime referencing technique using a direct current-to-digital converter architecture designed for energy-efficient wearables. The proposed design achieves 0.15-nA/cnt resolution over a 30-*μ*A input range at 88-*μ*W power consumption. With the proposed I-DAC current-scaling technique, the system maintains a luminescence ratio error of ≤0.5% across a wide input range.

## I. INTRODUCTION

RESPIRATORY parameters, such as the pressure of arterial oxygen (PaO_2_) and carbon dioxide (PaCO_2_) are critical indicators of pulmonary function and overall health [1]. Abnormalities in these metrics are associated with a wide range of respiratory disorders, including chronic obstructive pulmonary disease (COPD), which affects millions globally and represents a major cause of disease and mortality worldwide [2]. COPD and similar conditions often involve impaired gas exchange, increasing the likelihood of respiratory depression caused by carbon dioxide (CO_2_) accumulation [3]. Therefore, accurate and timely monitoring of PaCO_2_ is essential for detecting ventilation inefficiencies.

The clinical gold standard for PaCO_2_ measurement is arterial blood gas (ABG) analysis; however, ABG sampling is invasive, painful, and provides only intermittent snapshots of a patient’s ventilatory status [4]. Furthermore, ABG testing is confined to clinical settings, limiting its utility for continuous monitoring. These limitations highlight the need for technologies that enable continuous, noninvasive, and remote PaCO_2_ tracking, providing timely alerts for acute respiratory deterioration and guiding treatment outside the hospital environment [5].

Transcutaneous CO_2_ monitoring offers a noninvasive alternative for assessing PaCO_2_ by measuring the partial pressure of CO_2_ that diffuses through the skin (PtcCO_2_) [6]. PtcCO_2_ is strongly correlated with PaCO_2_ making it a reliable surrogate for ABG [7]. Conventional PtcCO_2_ sensors rely on electrochemical techniques that, while accurate, are constrained by bulky bedside monitors and high-power heating elements. These heaters are required to approximate transcutaneous measurements to arterial values and to reduce stabilization time and signal variability [8]. These limitations make traditional systems unsuitable for long-term or mobile use.

For wearable health applications, energy efficiency is critical: continuous monitoring must maintain clinical accuracy while minimizing power consumption to enable multiday operation on compact batteries. This challenge drives the need for highly integrated, low-power architectures tailored for wearable respiratory monitoring. Although several efforts have explored miniaturized PtcCO_2_ sensors [5], all reported implementations are based on discrete designs, which inherently lack the energy efficiency and scalability achievable with integrated circuit solutions.

A preliminary version of this work was presented in [9], where we introduced the first integrated luminescence-based transcutaneous CO_2_ sensing system implemented in a 180-nm CMOS process, leveraging the time-domain dual lifetime referencing technique [10]. Key innovations included: 1) high-speed luminescence capture and processing through a single detector and signal path, eliminating the need for complex optics, secondary reference circuits, and high-speed electronics; 2) a front-end based on a modified-delta-sigma (ΔΣ) modulator with direct current-to-digital conversion (CDC), removing the transimpedance amplifier (TIA) stage; and 3) an I-DAC-based current scaling approach that reduces hardware complexity by eliminating multiple comparators.

In this extended work, we introduce several additional technical contributions: 1) a comprehensive analysis of the time-domain dual lifetime referencing approach; 2) a detailed description of energy-efficient design strategies at both circuit and system levels—including: i) a direct current-to-digital converter that removes the need for an amplifier stage; ii) a current-scaling I-DAC that enhances sensitivity and dynamic range (DR) that supports lower excitation levels; iii) duty-cycled operation; and iv) a luminescent sensing approach that eliminates the heater; 3) an analysis of error sources along with corresponding mitigation techniques; 4) additional measurement results demonstrating the benefits of the proposed current-scaling approach; and 5) an updated human subject study comparing our system’s performance with that of a commercial PtcCO_2_ monitor.

The remainder of this article is structured as follows. Section II provides the underlying theory of the time-domain dual lifetime referencing method along with a detailed explanation of the sensing principles. Section III presents the system architecture, describing the methods employed for energy-efficient operation and error mitigation. Section IV discusses the experimental characterization, including bench-top gas measurements and human subject testing, to validate circuit performance. Section V concludes this article.

## II. SYSTEM OVERVIEW

### A. LUMINESCENCE SENSING

**FIGURE 1.**
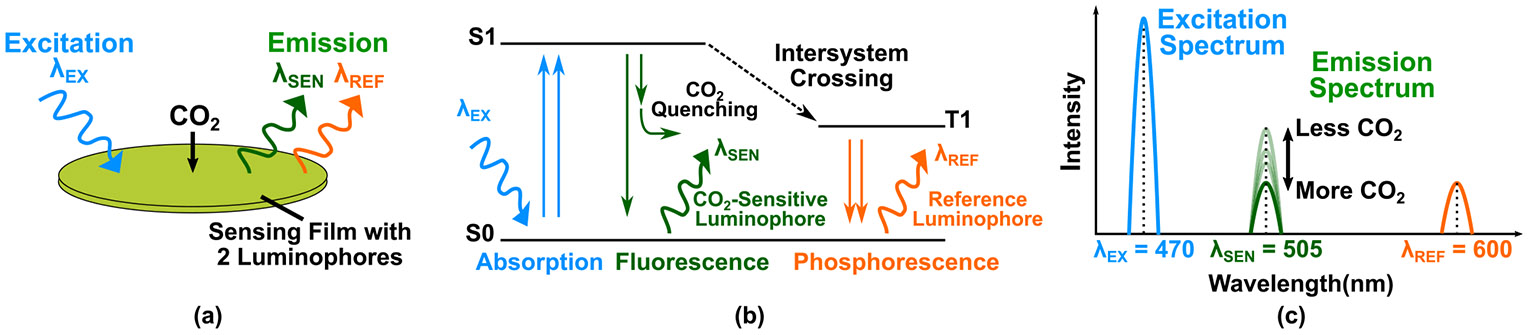
(a) Illustration of the sensing film, (b) simplified electronic state diagram, and (c) sensing film’s luminescence spectra.

We employed a CO_2_-sensitive luminescent film (CO_2_ Sensor Spot CD1T, PreSens Precision Sensing GmbH), illustrated in Fig. 1(a), for measuring the partial pressure of CO_2_ hereafter referred as the sensing film. The sensing film comprises two distinct types of atomic groups known as luminophores, namely, the CO_2_-sensitive luminophores and the reference luminophores. When these luminophores are excited by higher energy photons (*λ*_EX_ ≈ 470 nm), their electrons move from the ground state (S0) to an excited state (S1), as shown in Fig. 1(b). The electrons from the CO_2_-sensitive luminophore transition directly back to S0, resulting in photon emission known as fluorescence. In contrast, the electrons from the reference luminophore return to S0 via a triplet state (T1), leading to photon emission referred to as phosphorescence. As the electrons from both CO_2_-sensitive and reference luminophores return to S0, they emit photons at longer wavelengths (*λ*_SEN_ ≈ 505 nm and *λ*_REF_ ≈ 600 nm) as demonstrated in the emission spectrum of the film, given in Fig. 1(c). The emission of the CO_2_-sensitive luminophore is reduced in the presence of CO_2_, leading to a decrease in both intensity $\left(I_{\text{SEN}}\right)$ and lifetime $\left(\tau_{\text{SEN}}\right)$ as CO_2_ partial pressure (PCO_2_) increases. This relationship is described by the Stern-Volmer equation [11]

(1) $$\frac{I_{\text{SEN}0}}{I_{\text{SEN}}}=\frac{\tau_{\text{SEN}0}}{\tau_{\text{SEN}}}=1+K_{\text{SV}}\times {\text{PCO}}_{2}$$

where $I_{\text{SEN}0}$ and $\tau_{\text{SEN}0}$ represent the intensity and lifetime of the CO_2_-sensitive luminophore in the absence of CO_2_, and $K_{\text{SV}}$ is the Stern-Volmer constant. Rearranging (1), $I_{\text{SEN}}$ is expressed as

(2) $$I_{\text{SEN}}=\frac{I_{\text{SEN}0}}{1+K_{\text{SV}}\times {\text{PCO}}_{2}}.$$

$I_{\text{SEN}0}$ depends on the excitation intensity, $I_{\text{EXT}}$, and the responsivity of the CO_2_-sensitive luminophore, $S_{\text{SEN}}$. Substituting these terms gives

(3) $$I_{\text{SEN}}=\frac{I_{\text{EXT}}\times S_{\text{SEN}}}{1+K_{\text{SV}}\times {\text{PCO}}_{2}}.$$

Unlike the CO_2_-sensitive luminophore, the reference luminophore exhibits an intensity, $I_{\text{REF}}$, and lifetime, $\tau_{\text{REF}}$, that are insensitive to the variations in PCO_2_. Given that both luminophores are exposed to the same excitation intensity, $I_{\text{EXT}}$, the reference intensity is

(4) $$I_{\text{REF}}=I_{\text{REF}0}=I_{\text{EXT}}\times S_{\text{REF}}$$

where $S_{\text{REF}}$ is the responsivity of the reference luminophore.

### B. TIME-DOMAIN DUAL LIFETIME REFERENCING THEORY

A key difference between the CO_2_-sensitive and reference luminophores is their lifetimes. The CO_2_-sensitive luminophore has a lifetime measured in nanoseconds (ns), whereas the reference luminophore has a lifetime measured in microseconds (*μ*s). When the sensing film is excited with a *μ*s-long pulse of blue light, both luminophores contribute to the corresponding luminescence while the excitation is on. However, when the excitation is turned off, the CO_2_-sensitive luminophore decays rapidly in the ns time frame due to its short lifetime, and its contribution to the luminescence is insignificant compared to the contribution of the reference luminophore, which decays in *μ*s.

**FIGURE 2.**
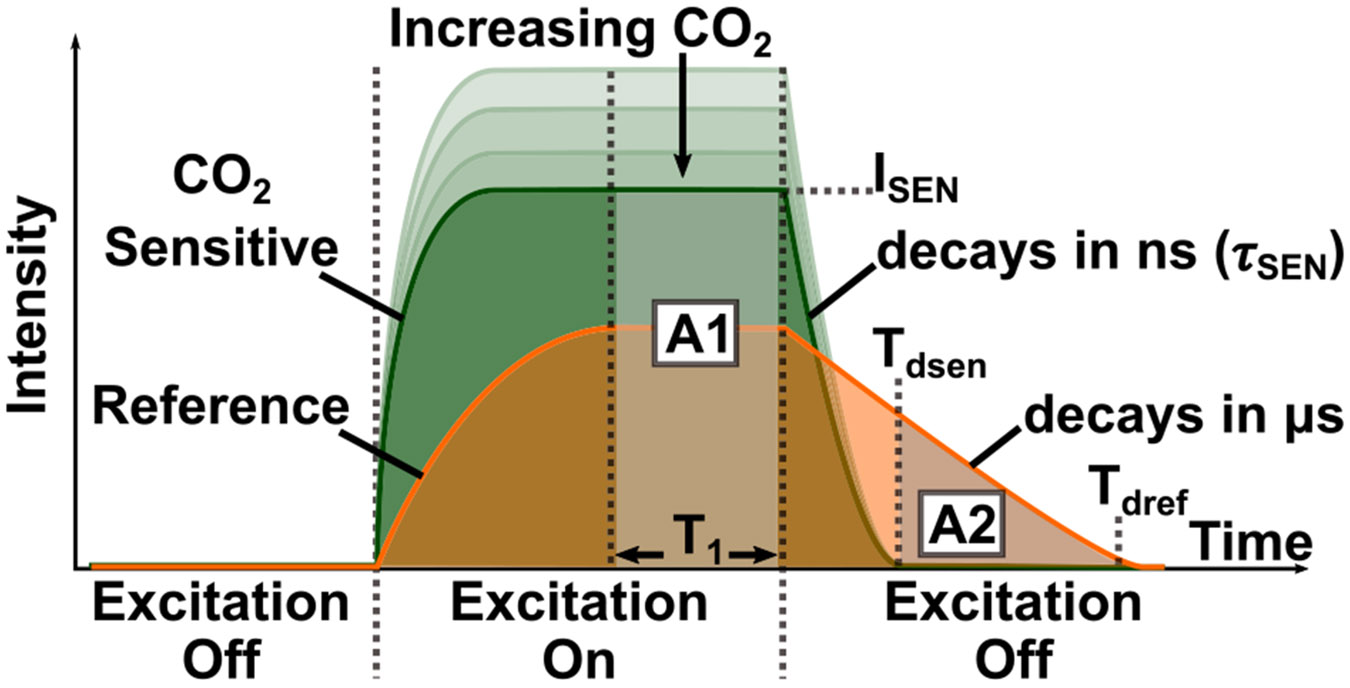
Time-domain luminescence response of the sensing film when excited by a pulse of blue light.

The luminescence response of the sensing film is depicted in Fig. 2. The overall luminescence from the CO_2_-sensitive and reference luminophores, denoted as $A_{1}$, can be represented by the equation

(5) $$A_{1}=(I_{\text{SEN}}+I_{\text{REF}})\times T_{1}$$

where $T_{1}$ indicates the excitation duration after the responses of both luminophores have stabilized, and $I_{\text{SEN}}$ and $I_{\text{REF}}$ represent the luminescence intensities of the CO_2_-sensitive and reference luminophores, respectively.

When the excitation is turned off, the total luminescence, $A_{2}$, can be expressed by disregarding the luminescence of the CO_2_-sensitive luminophore, as it is significantly smaller than that of the reference luminophore due to its much shorter decay time

(6) $$A_{2}=\int_{T_{\text{dsen}}}^{T_{\text{dref}}}I_{\text{REF}}\times e^{\frac{-t}{\tau_{\text{REF}}}}dt.$$

Given that $T_{\text{dref}}$ is much greater than $T_{\text{dsen}}$, this equation simplifies to

(7) $$A_{2}\approx I_{\text{REF}}\times \tau_{\text{REF}}.$$

The ratio of luminescence $A_{1}$ to $A_{2}$, referred to as the luminescence ratio (LR), is given by

(8) $$\text{LR}=\frac{A_{1}}{A_{2}}=\frac{(I_{\text{SEN}}+I_{\text{REF}})\times T_{1}}{I_{\text{REF}}\times \tau_{\text{REF}}}$$

which can also be expressed as

(9) $$\text{LR}=\frac{A_{1}}{A_{2}}=\left(\frac{I_{\text{SEN}}}{I_{\text{REF}}}+1\right)\times \frac{T_{1}}{\tau_{\text{REF}}}.$$

Substituting $I_{\text{SEN}}$ and $I_{\text{REF}}$ from (3) and (4) in (9)

(10) $$\text{LR}=\left(\frac{I_{\text{EXT}}\times S_{\text{SEN}}}{1+K_{\text{SV}}\times {\text{PCO}}_{2}}\times \frac{1}{I_{\text{EXT}}\times S_{\text{REF}}}+1\right)\times \frac{T_{1}}{\tau_{\text{REF}}}$$

and rearranging

(11) $$\text{LR}=\left(\frac{S_{\text{SEN}}}{S_{\text{REF}}}\times \frac{1}{1+K_{\text{SV}}\times {\text{PCO}}_{2}}+1\right)\times \frac{T_{1}}{\tau_{\text{REF}}}.$$

Note that $I_{\text{EXT}}$ is canceled out, therefore LR inherently immune to variations in the excitation Solving (11) for PCO_2_ gives

(12) $${\text{PCO}}_{2}=\left(\frac{1}{\text{LR}\times \frac{\tau_{\text{REF}}}{T_{1}}-1}\times \frac{S_{\text{SEN}}}{S_{\text{REF}}\times K_{\text{SV}}}\right)-\frac{1}{K_{\text{SV}}}.$$

In (12), $\tau_{\text{REF}}$ and $K_{\text{SV}}$ are fixed constants, while $T_{1}$ is a user-defined system parameter. The responsivities $S_{\text{SEN}}$ and $S_{\text{REF}}$ remain stable in the short term but can be affected by environmental conditions such as temperature and humidity, and may drift over time due to photobleaching, prolonged excitation, and drying of the sensing film [12]. Consequently, LR exhibits a direct inverse relationship with PCO_2_. This method, known as time-domain dual lifetime referencing (t-DLR), yields results that are unaffected by variations in $I_{\text{EXT}}$, offering a distinct benefit over conventional intensity-based measurements, which are sensitive to such changes in $I_{\text{EXT}}$ [13].

## III. SYSTEM ARCHITECTURE

### A. CIRCUIT-LEVEL APPROACHES FOR ENERGY EFFICIENCY

#### 1) DIRECT CURRENT-TO-DIGITAL CONVERSION WITHOUT AN AMPLIFIER

The sensing film is illuminated with short light pulses to reduce power consumption and mitigate photobleaching, thereby extending its lifespan. Consequently, the photocurrent delivered to the luminescence sensing system consists of a brief pulse, lasting a few *μ*s, followed by a luminescence decay of comparable duration. Typically, a TIA with resistive feedback is employed as the first stage to convert this photocurrent into a voltage before digitization by an analog-to-digital converter (ADC) for further processing [12], [13], [14], [15], [16]. However, these architectures struggle with such short pulses and decays due to the bandwidth limitations of the TIA [17]. For a resistive TIA (R-TIA), the bandwidth is constrained by

(13) $$R_{F}\leq \frac{\text{GBW}}{2\pi \times C_{IN}\times f_{BW}^{2}}$$

where $R_{F}$ is the feedback resistor, GBW is the TIA’s gain-bandwidth product, $C_{\text{IN}}$ is the input capacitance, including photodiode (PD) junction and parasitic capacitance, and fBW is the TIA bandwidth. Achieving sufficient gain to amplify photocurrents in the nA-*μ*A range to mV-V levels requires a large $R_{F}$, which further limits bandwidth. In addition, larger resistors pose practical challenges in CMOS design due to area constraints. Moreover, $R_{F}$ contributes to input-referred noise; increasing $R_{F}$ reduces noise but further narrows the closed-loop bandwidth.

An alternative is the capacitive TIA (C-TIA), which replaces the feedback resistor with a noiseless feedback capacitor $\left(C_{F}\right)$ [18]. However, this design is susceptible to saturation, unless it is addressed with additional circuitry, such as switch capacitors that prevent saturation by charge injection at given times [19], and requires a reset switch across $C_{F}$ to periodically discharge the capacitor and provide a DC feedback path. Reset schemes can restrict bandwidth, and while continuous reset methods preserve bandwidth, they compromise linearity, necessitating the use of a differentiating amplifier to restore linearity at the cost of increased power consumption [20].

**FIGURE 3.**
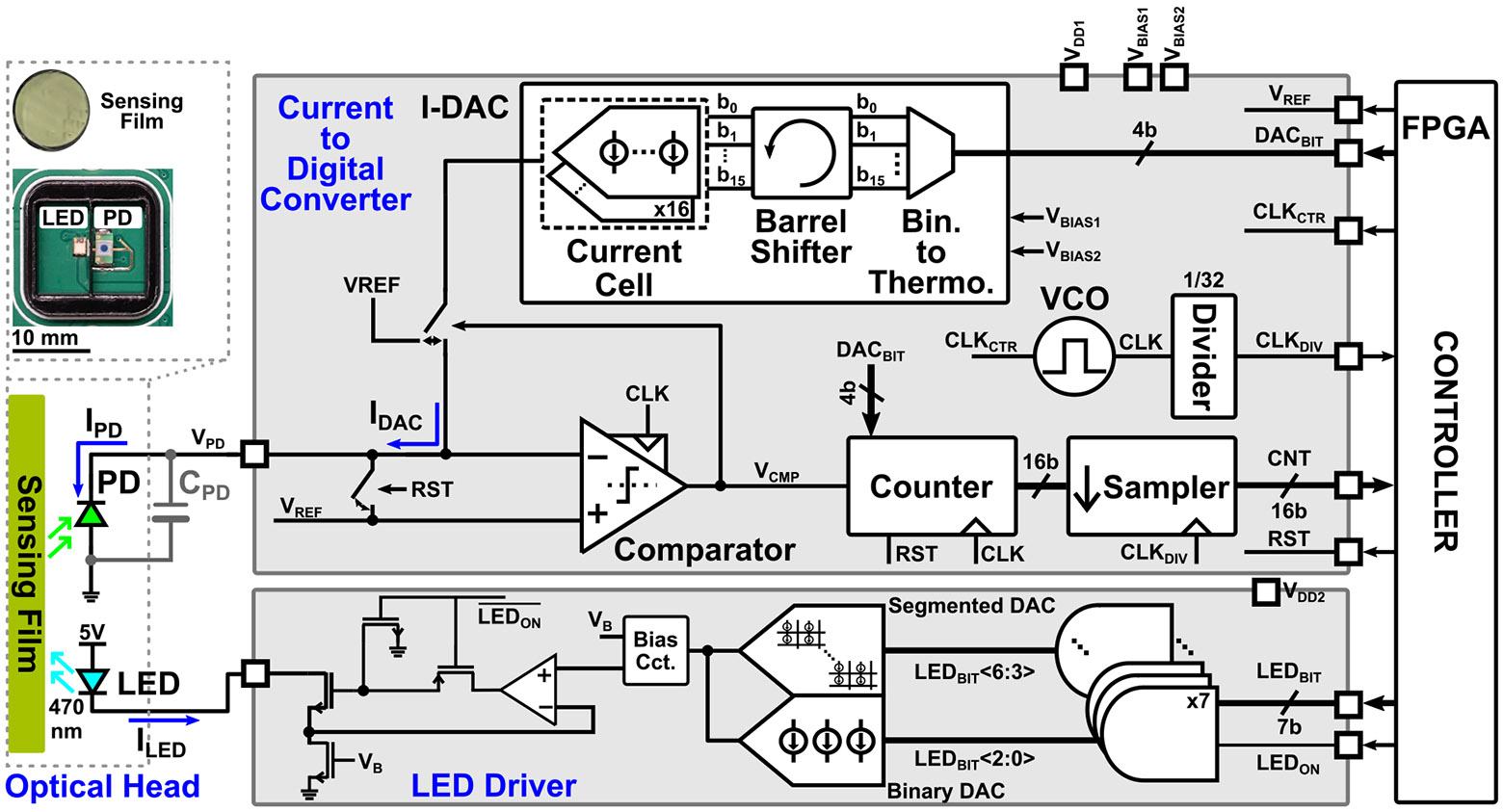
System diagram, featuring a current-to-digital front end, an LED driver, and an optical head.

As depicted in Fig. 3, the proposed architecture directly feeds the photocurrent into a modified-ΔΣ modulator. Different from a conventional ΔΣ modulator, which targets waveform reconstruction, the proposed modified-ΔΣ architecture is designed to solely integrate the input charge. The proposed design utilizes the PD’s junction capacitance as the integration element, eliminating the need for an additional integration stage or gain. This approach removes the requirement for a TIA and enables a direct CDC scheme [21]. Moreover, since waveform reconstruction is not required, the decimation filter is omitted.

In the proposed CDC architecture, the luminescence emitted by the sensing film is captured by a PD, generating a photocurrent $I_{\text{PD}}$ that modulates the PD bias voltage $V_{\text{PD}}$. To maintain a stable $V_{\text{PD}}$ under dynamic variations in sensor luminescence, a current digital-to-analog converter (I-DAC) injects compensating current into the junction capacitance $C_{\text{PD}}$. The printed circuit board (PCB) and wire-bond parasitics contribute an estimated capacitance of approximately 0.2 pF, which is negligible compared to the typical $C_{\text{PD}}$ of 3pF [22]. The I-DAC is digitally controlled through 4-bit signals (${\text{DAC}}_{\text{BIT}}$) provided by an FPGA. These control bits are first converted from binary to thermometer code to ensure monotonicity and then processed by a barrel shifter. The barrel shifter shifts the bit pattern by one position each cycle to suppress deterministic mismatch errors. Averaging across multiple cycles ensures that these mismatches do not accumulate in the generated current. The shifted thermometer code drives an array of unit current cells to synthesize the I-DAC output current.

A StrongArm latch comparator, shown in Fig. 6(a), monitors $V_{\text{PD}}$ against a reference voltage $V_{\text{REF}}$ and regulates the I-DAC switching activity. Each comparator decision corresponds to a discrete current injection event, and the resulting output signal $\left(V_{\text{CMP}}\right)$ is accumulated by a 16-bit synchronous counter. This count represents the integrated photocurrent and is subsequently downsampled to match the post-processing clock domain $\left({\text{CLK}}_{\text{DIV}}\right)$. The counter serves only to accumulate information from the output of the modified ΔΣ, and is not inside the feedback loop. The comparator output, $V_{\text{CMP}}$ directly controls I-DAC current switching, providing a 1^st^-order feedback which is inherently stable. To eliminate reliance on an external clock source, an on-chip voltage-controlled oscillator (VCO) generates a high-frequency clock, while a programmable clock control block $\left({\text{CLK}}_{\text{CTR}}\right)$ adjusts the oscillator frequency $\left(f_{\text{CLK}}\right)$, and, hence the resolution. ${\text{CLK}}_{\text{DIV}}$ drives the digital controller, ensuring synchronized operation across the system.

The supply rails for the CDC and LED driver, denoted as $V_{\text{DD1}}$ and $V_{\text{DD2}}$, are provided through external low-dropout regulators (LDOs). The bias voltages for the I-DAC current cell, $V_{\text{BIAS}1}$ and $V_{\text{BIAS}2}$, are generated by an external dual-channel DAC. In addition, $V_{\text{REF}}$ and ${\text{CLK}}_{\text{CTR}}$ are sourced from the FPGA’s integrated dual-channel DAC.

#### 2) CURRENT-SCALING I-DAC ENABLING LOW-INTENSITY EXCITATION

An LED serves as the excitation source; however, the LED driver typically contributes significantly to the system’s power consumption [12], [23]. To address this, we introduce a current-scaling I-DAC technique that enhances CDC sensitivity while preserving a wide DR. Higher sensitivity in the CDC reduces the required LED excitation intensity, thereby lowering the power consumption of the LED driver and improving overall energy efficiency.

**FIGURE 4.**
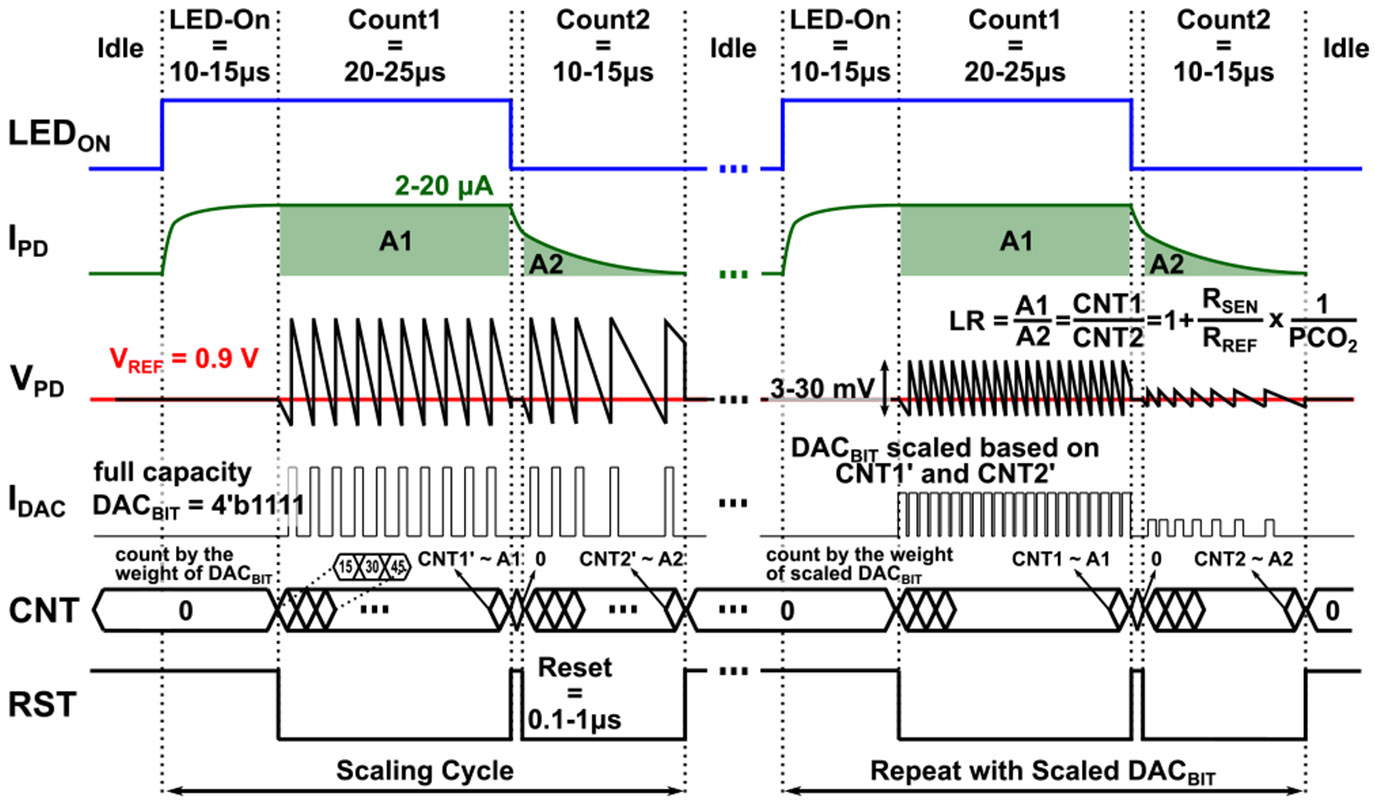
Timing diagram of the I-DAC current scaling technique.

The operating principle of the proposed current-scaling method is illustrated in Fig. 4. The system begins in an “Idle” state, with $V_{\text{PD}}$ biased to $V_{\text{REF}}$. After this state, the LED Driver is activated (“LED-On” state) while other components remain idle, allowing the sensing film’s luminescent response to stabilize within 10–15 *μ*s. During the final 0.5 *μ*s of the “LED-On” phase, the remaining circuitry is activated to facilitate the start-up time. The system then transitions to the “Count1” state, where the I-DAC operates at full scale, and the counter accumulates current injections. The resulting count, CNT1’, corresponds to the area under the $I_{\text{PD}}$ curve $A_{1}$, representing the combined emission from CO_2_-sensitive and reference luminophores. In the subsequent “Count2” state, the I-DAC again operates at full scale, and CNT2’ reflects the decay curve area of the reference luminophores $\left(A_{2}\right)$. The cycle concludes by returning to the “Idle” state. The FPGA then updates the ${\text{DAC}}_{\text{BIT}}$ value using a look-up table indexed by the counter outputs CNT1’ and CNT2’. This scaled ${\text{DAC}}_{\text{BIT}}$ setting is then held constant for the next 100 measurement cycles. Over these cycles, the outputs CNT1 and CNT2 are averaged to compute the LR. The total conversion time from $I_{\text{PD}}$ to LR for 100 samples, including the scaling cycle, is approximately 15.5 ms. It is important to note that ${\text{DAC}}_{\text{BIT}}$ adjustments are implemented as long-term updates rather than per-cycle modifications, ensuring that the feedback loop remains uninterrupted. The loop closure is maintained through the comparator output.

The proposed current-scaling technique achieves high sensitivity while providing flexibility to extend the DR of the input current, thereby enhancing system robustness under varying optical path and luminophore conditions. This approach not only reduces LED driver power consumption through improved CDC sensitivity but also preserves a wide DR, accommodating variations such as changes in the film’s responsivity caused by photobleaching during extended operation. Furthermore, the current-scaling scheme improves conversion accuracy without requiring multiple comparators, thereby reducing both area and power overhead.

### B. SYSTEM-LEVEL APPROACHES FOR ENERGY EFFICIENCY

#### 1) DUTY CYCLING

Direct CDC architectures are inherently more suitable for duty-cycled operation as they eliminate the TIAs and other analog front-end blocks present in traditional designs, which require continuous biasing. Beyond this architectural advantage, respiratory parameters change slowly; therefore, PtcCO_2_ monitors typically sample at intervals of several seconds [24], [25]. This enables aggressive duty cycling since capturing a single LR measurement only takes a few *μ*s. To minimize power while maintaining measurement accuracy, the excitation pulse must be short yet sufficient to fully excite the sensing film. An excitation duration of 30–40 *μ*s was identified as optimal [12], ensuring adequate luminophore excitation while limiting energy consumption. Based on these considerations, the proposed system operates at a 1% duty cycle to maximize energy efficiency. In practice, this means the system remains active only during the LED-On, Count1, and Count2 states for 1% of the time, while spending the remaining time in the Idle state. During the Idle state, the LED driver is disabled by pulling ${\text{LED}}_{\text{ON}}$ low, and the external LDOs, supplying $V_{\text{DD1}}$ and $V_{\text{DD2}}$, are turned off.

#### 2) TRANSCUTANEOUS MEASUREMENT WITHOUT A HEATER

CO_2_ diffusion through the skin is slow; therefore, conventional benchtop electrode-based PtcCO_2_ monitors employ heaters to elevate the skin temperature to 42 °C–44 °C, enhancing local arterialization and accelerating CO_2_ diffusion. Even with heating, these systems require approximately 5–10 min to stabilize, whereas without heating, stabilization can take up to 85 min [26]. The heaters typically consume power in the milliwatt range [24], [25]. In contrast, the proposed approach utilizes a luminescent sensing film, achieving a stabilization time of ~10 min without any heating, thereby maintaining comparable performance while significantly reducing power consumption.

### C. APPROACHES FOR MITIGATING ERROR SOURCES

The design objective was to achieve both high energy efficiency and resolution without sacrificing the DR. According to Food and Drug Administration guidelines, PtcCO_2_ monitors must provide a minimum resolution of 5 mmHg [27]. In this work, a target resolution of approximately 2.5 mmHg was selected, which is twice as stringent as the regulatory requirement. Based on prior gas testbench measurements, this corresponds to an LR resolution of about 0.5% [12]. Consequently, the system was designed to ensure an average LR error of 0.5%.

To quantify LR error, LR is first expressed in terms of system parameters. The total charge integrated on $C_{\text{PD}}$ during excitation, $Q_{1}$, equals the area $A_{1}$ under the luminescence curve in Fig. 2. Using (5) and substituting luminescence intensities $I_{\text{SEN}}$ and $I_{\text{REF}}$ with their corresponding photocurrents $I_{\text{PD(SEN)}}$ and $I_{\text{PD(REF)}}$, $Q_{1}$ is expressed as

(14) $$Q_{1}=(I_{\text{PD(SEN)}}+I_{\text{PD(REF)}})\times T_{1}.$$

Similarly, the integrated charge after excitation, $Q_{2}$, corresponds to the area, $A_{2}$, under the decay curve. From (7), $Q_{2}$ can be expressed as

(15) $$Q_{2}\approx I_{\text{PD(REF)}}\times \tau .$$

Thus, LR is calculated as

(16) $$\frac{Q_{1}}{Q_{2}}=\left(\frac{I_{\text{PD(SEN)}}}{I_{\text{PD(REF)}}}+1\right)\times \frac{T_{1}}{\tau }.$$

The CDC injects a balancing current, $I_{\text{DAC}}$, for a duration proportional to counts CNT1 and CNT2, such that

(17) $$Q_{1}=\text{CNT}1\times (I_{\text{DAC}}\times T_{\text{CLK}}),Q_{2}=\text{CNT}2\times (I_{\text{DAC}}\times T_{\text{CLK}})$$

substituting into (16) and rearranging the equation gives the LR as

(18) $$\frac{I_{\text{PD(SEN)}}}{I_{\text{PD(REF)}}}=\left(\frac{\text{CNT}1}{\text{CNT}2}\times \frac{\tau }{T_{1}}\right)-1.$$

#### 1) CONVERSION ERROR

In the proposed architecture, the dominant contribution to what would traditionally be considered quantization error arises only in the final conversion step. Transient errors introduced during the integration phase are naturally corrected by the modulator in later cycles. Because no waveform reconstruction is required, these intermediate deviations neither accumulate nor propagate. The integration serves as an averaging mechanism, allowing such fluctuations to cancel out over multiple cycles. As a result, the effective quantization error is limited to 1 LSB, defined as the ratio of a single count to the full-scale count. The conversion errors associated with CNT1 and CNT2 are represented by $\epsilon_{1}$ and $\epsilon_{2}$, respectively. This error is inversely proportional to CNT1 and CNT2, implying that increasing $T_{1}$ or the clock frequency $\left(T_{\text{CLK}}\right)$ reduces error. Conversely, the number of DAC bits, ${\text{DAC}}_{\text{BIT}}$, is proportional to the conversion error. The proposed current-scaling algorithm mitigates this by reducing ${\text{DAC}}_{\text{BIT}}$ for smaller photocurrents. The conversion errors for CNT1 and CNT2 can be approximated as

(19) $$\epsilon_{1}∼{\text{DAC}}_{\text{BIT}}\times \frac{T_{\text{CLK}}}{T_{1}},\epsilon_{2}∼{\text{DAC}}_{\text{BIT}}\times \frac{T_{\text{CLK}}}{\tau }.$$

In the worst case, errors add

(20) $$\epsilon_{1}+\epsilon_{2}∼{\text{DAC}}_{\text{BIT}}\times T_{\text{CLK}}\times \left(\frac{1}{T_{1}}+\frac{1}{\tau }.\right)$$

**FIGURE 5.**
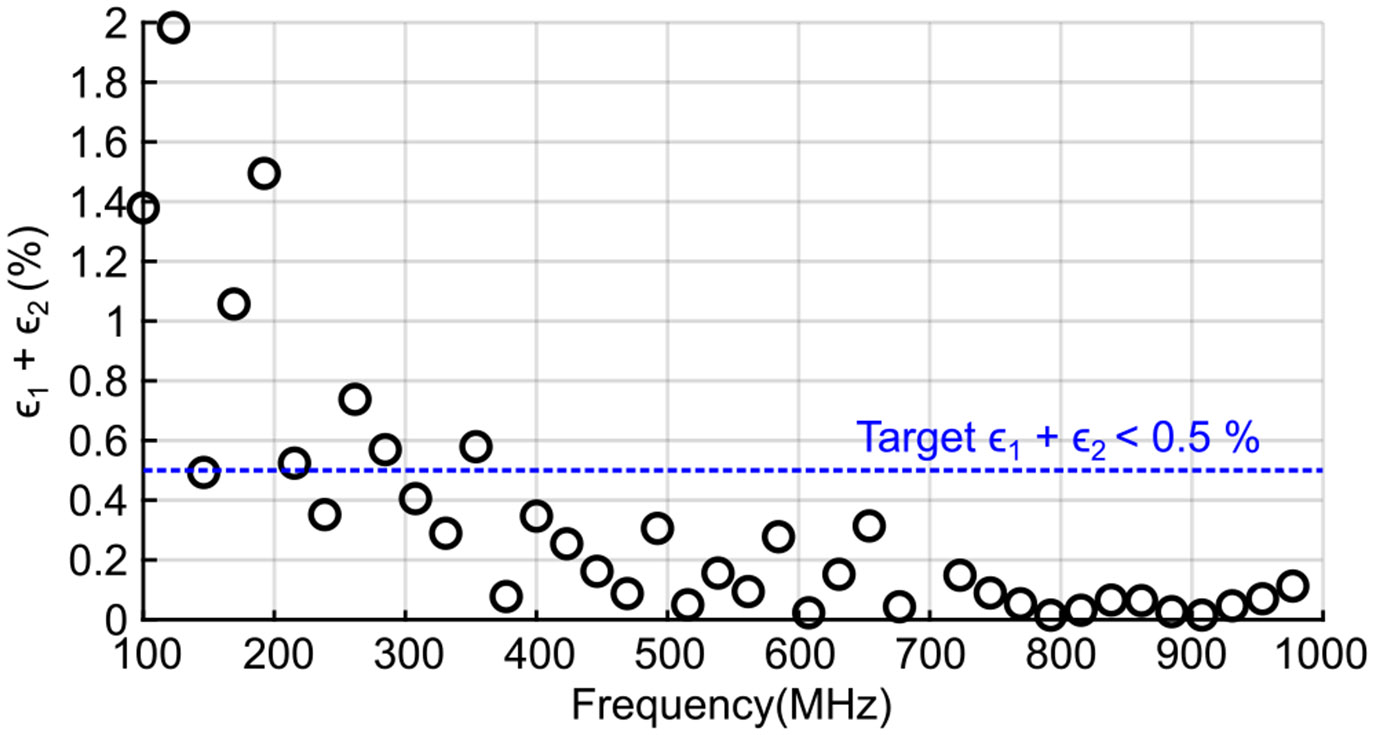
Behavioral simulation results demonstrating the total conversion error $\left(\epsilon_{1}+\epsilon_{2}\right)$ versus the clock frequency.

Since $T_{1}$ is on the order of tens of *μ*s while τ is only a few *μ*s, $\epsilon_{2}$ dominates. Increasing $T_{1}$ improves accuracy but also raises power consumption due to a longer excitation period. Consequently, increasing the clock frequency provides the primary lever for reducing conversion errors, albeit at the cost of higher power consumption. Therefore, clock optimization should carefully balance accuracy and power by quantifying the associated power cost. An optimal clock frequency is then selected to satisfy the 0.5% error target. To determine this operating point, a behavioral simulation was performed using realistic parameters: $I_{\text{PD(SEN)}}$ and $I_{\text{PD(REF)}}$ set to 500 nA, *τ* = 1 *μ*s, *T*_1_ = 30 *μ*s, and I_DAC(LSB)_ = 500 nA. As shown in Fig. 5, the error falls below the 0.5% target at frequencies above approximately 300 MHz. Based on these results, the system clock frequency $f_{\text{CLK}}$ was chosen as 320 MHz, balancing accuracy and power consumption.

#### 2) COMPARATOR OFFSET

Another source of error arises from the comparator input offset, denoted as $\Delta V_{\text{off}}$. This offset introduces an additional component to the integrated charge in (14) and (15), expressed as

(21) $$\epsilon_{Q}=C_{\text{PD}}\times \Delta V_{\text{off}}.$$

To minimize this offset, the cross-coupled transistor pairs (M_3_–M_6_) are carefully sized to reduce mismatch while maintaining comparator speed with negligible performance degradation. A simulated post-layout input offset of 25 mV was measured. This offset, $\Delta V_{\text{off}}$ translates to a charge error, $\epsilon_{Q}$, of 75 fC, assuming C_PD_ = 3 pF [22]. The impact of this error becomes more significant in the second integration phase, $A_{2}$, where the accumulated charge $Q_{2}$ is smaller. The corresponding current error during the decay phase can be expressed as

(22) $$\epsilon_{I}=\epsilon_{Q}\times \tau .$$

For a time constant *τ* = 1 *μ*s, the resulting $\epsilon_{I}$ is 75 nA. Consequently, the comparator offset introduces a more significant error when the input current is in the sub-*μ*A range. However, since this offset remains constant across operations, it behaves as a DC error and can be removed during post-processing.

#### 3) COMPARATOR INPUT-REFERRED NOISE

**FIGURE 6.**
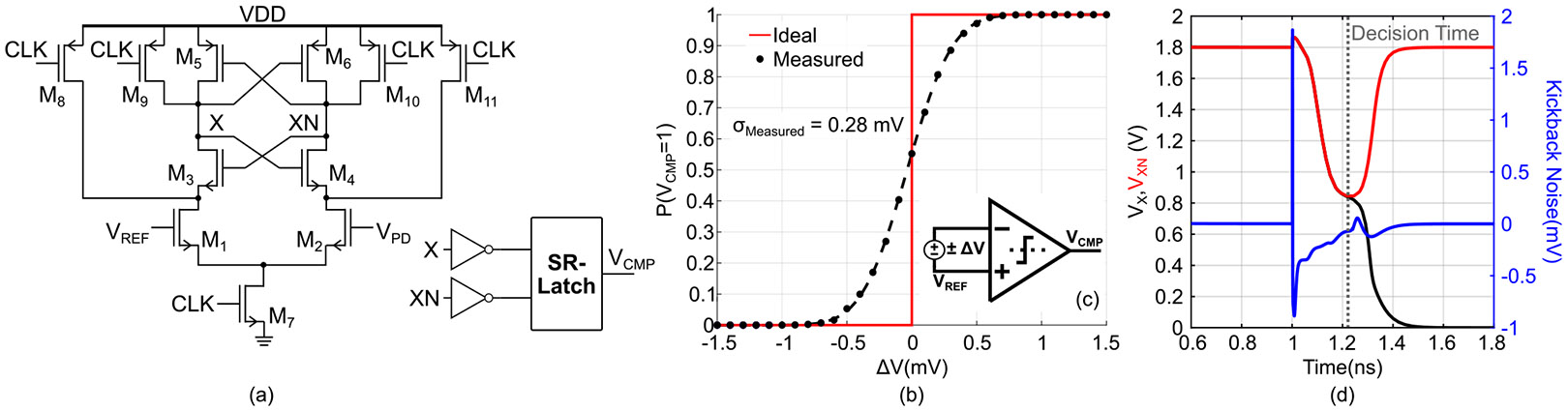
(a) Schematic of the comparator. (b) Probability density of the comparator output $\left(V_{\text{CMP}}\right)$ with a measured standard deviation $\left(\sigma_{\text{Measured}}\right)$ of 0.28 mV_rms_. (c) Test setup for simulating the input-referred noise. (d) Timing of the kickback noise.

The comparator’s input-referred noise directly influences the integration node voltage, $V_{\text{PD}}$ and therefore must be examined carefully. To evaluate the input-referred noise the comparator was simulated in the configuration shown in Fig. 6(c), where a small input differential ΔV was applied. The ΔV was swept from −1.5 to 1.5 mV, and the comparator output, $V_{\text{CMP}}$ was recorded over 1000 clock cycles for each value. The probability density of the comparator output being high (VDD) is shown in Fig. 6(b). Ideally, this probability distribution should exhibit a step transition, however it is skewed due to input-referred noise. The standard deviation of the probability density curve gives the total root-mean-square (rms) referred to the input [28], which was found to be 0.28 mV_rms_. This value indicates that the comparator’s input-referred noise contributes to the system error negligibly compared to its input offset.

#### 4) COMPARATOR KICKBACK NOISE

The last source of error from the comparator is the kickback noise. Fig. 6(d) illustrates the simulated waveforms of the comparator signals X and XN during the comparison phase, along with the corresponding kickback noise. Since the occurrence of the kickback noise is temporally separated from the comparator’s decision time, it does not interfere with the comparison process. Furthermore, in the CDC architecture, charge integration occurs over multiple cycles, and the injected charges due to kickback average out to zero over the integration period. Consequently, the kickback noise does not introduce any net charge error.

#### 5) CHARGE INJECTION THROUGH THE I-DAC SWITCH

**FIGURE 7.**
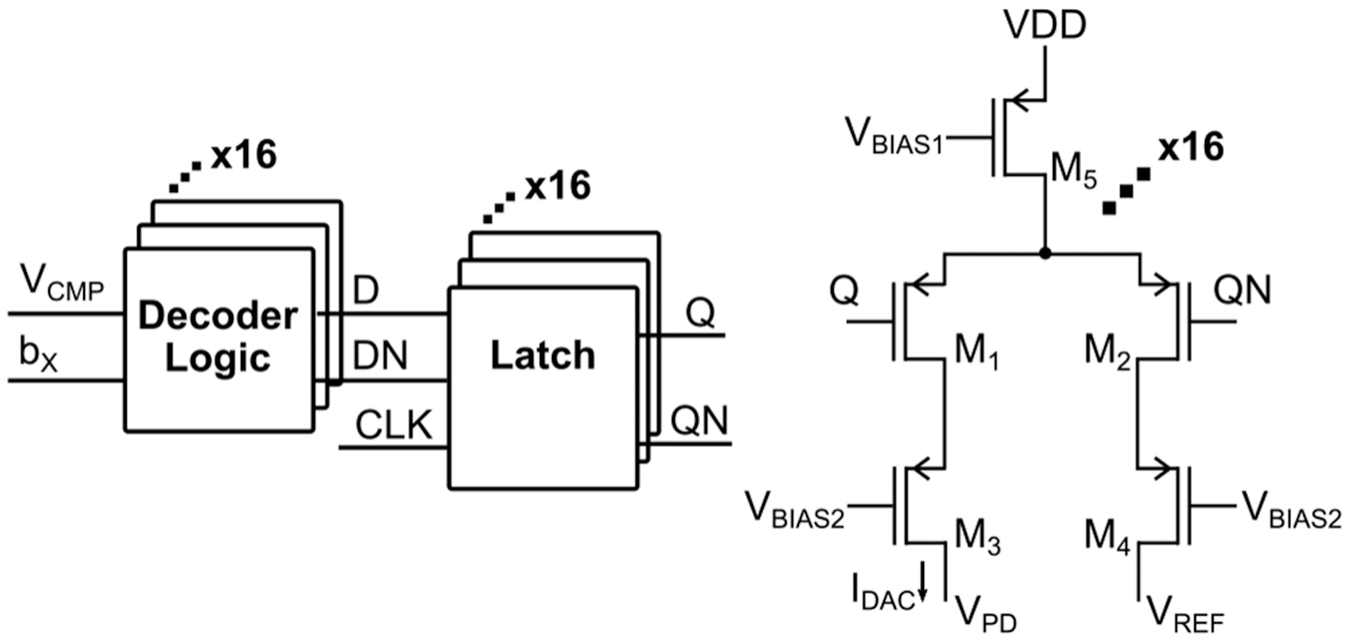
Schematic of the I-DAC current cell.

When the I-DAC current cell turns off, charge stored in the gate oxide of the I-DAC switch can be injected into C_PD_, introducing an additional error term in (14) and (15). This charge injection becomes increasingly significant at higher operating frequencies, where switching activity is more frequent. To suppress this effect, dummy transistors M_3_ and M_4_ are inserted between the I-DAC switch (M1) and node $V_{\text{PD}}$, as illustrated in Fig. 7, effectively canceling the injected charge.

#### 6) QUANTIZATION NOISE

The proposed CDC employs a modified ΔΣ architecture in which, unlike a conventional ΔΣ modulator, the quantization noise is not shaped out of the signal band. The quantization noise is directly related to the vertical excursions in $V_{\text{PD}}$ shown in Fig. 4; when multiplied by $C_{\text{PD}}$, these excursions correspond to the uncertainty in accumulated charge. The proposed I-DAC current scaling technique, reduces the size of the charge packets by scaling the I-DAC current according to the input photocurrent and correspondingly reduces the $V_{\text{PD}}$ excursions. This reduction in $V_{\text{PD}}$ swing lowers the quantization noise even in the absence of traditional ΔΣ noise shaping, thereby improving overall sensitivity.

Furthermore, noise sources that would typically be shaped out in a conventional design, such as the comparator’s input-referred noise, are minimized through careful circuit optimization. In the implemented system, $V_{\text{PD}}$ excursions range from 3–20 mV, whereas the comparator’s input-referred noise is only 0.28 mV_rms_, over an order of magnitude smaller. Consequently, the comparator’s input-referred noise has a slight impact on the overall performance. Although the modified-ΔΣ architecture does not benefit from quantization noise shaping, this limitation is offset by the I-DAC current scaling technique and careful noise-aware design. In return, the architecture achieves lower power consumption and a simpler analog front-end by avoiding the need for a TIA-based integrator.

## IV. MEASUREMENT RESULTS

**FIGURE 8.**
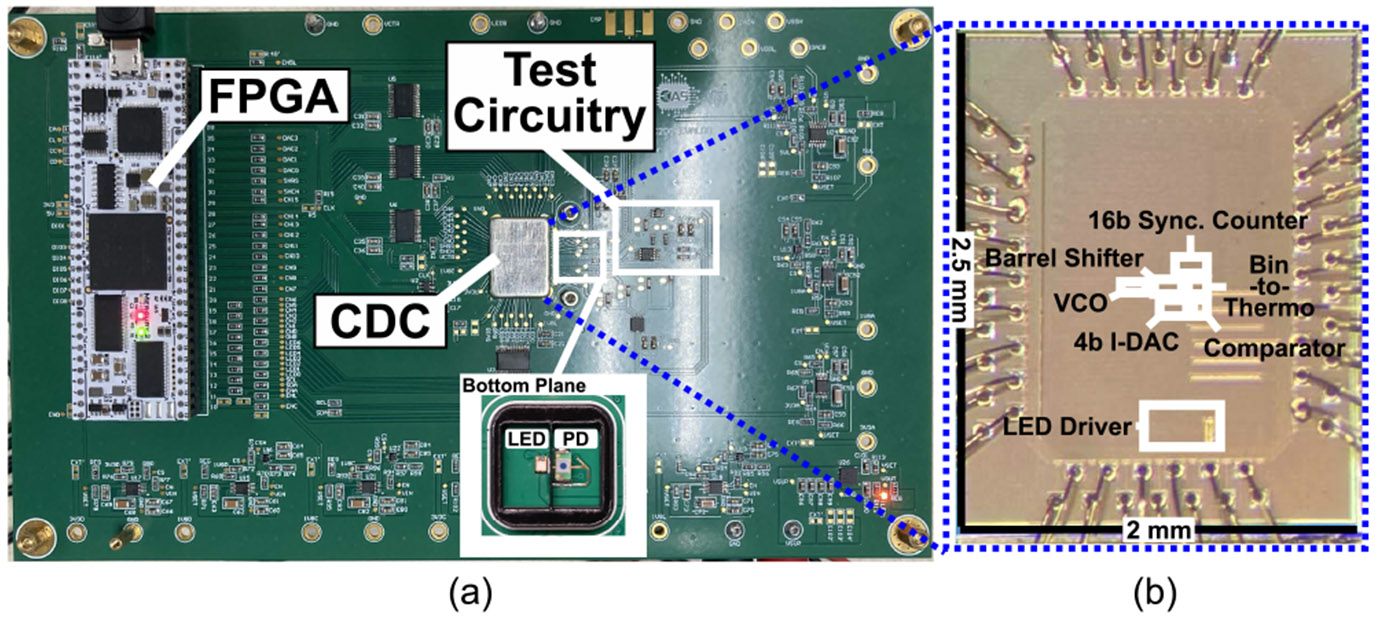
(a) Evaluation board and (b) and the micrograph of the CDC chip.

The CDC chip, shown in Fig. 3, was fabricated using the TSMC 180 nm MS/RF/G process. The evaluation board and the chip micrograph are presented in Fig. 8(a) and (b), respectively. The following subsections elaborate on the three primary sets of experiments conducted: 1) CDC chip characterization; 2) bench-top gas measurements; and 3) a human subject test.

### A. CHARACTERIZATION OF THE CDC

To characterize the CDC chip, a test current was sunk through the $V_{\text{PD}}$ input to emulate the PD’s current, using an external 7-bit current-steering DAC (DS4432, Maxim Integrated). To eliminate ambient light interference during measurements, the PD surface was covered with black tape, ensuring that the dark current of the PD, typically around 10 pA [22], is the only optical noise source.

**FIGURE 9.**
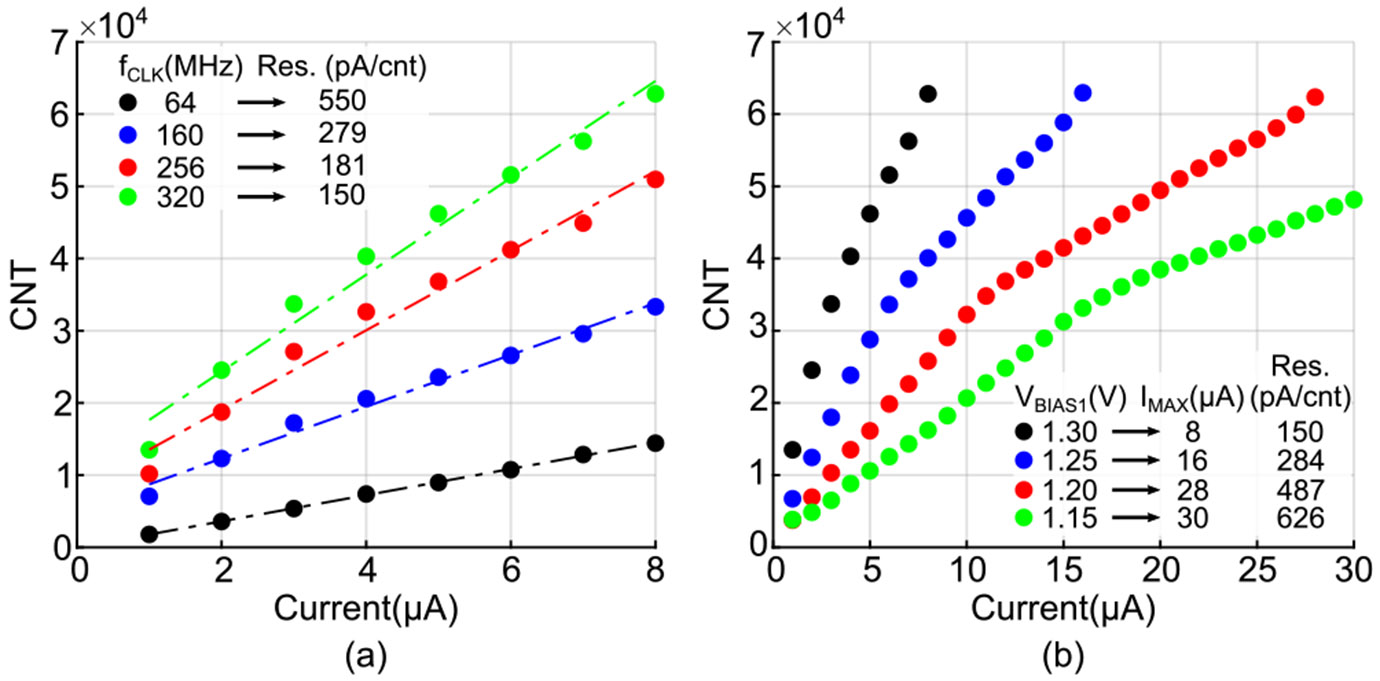
Input current versus the output of the CDC (a) at $V_{BIAS1}=1.3$ with different $f_{\text{CLK}}$ values, and (b) at $f_{\text{CLK}}=320$ with varying $V_{BIAS1}$ levels.

The operating frequency of the CDC, $f_{\text{CLK}}$, is adjustable via the on-chip VCO. Adjustable $f_{\text{CLK}}$ enables users to control the resolution of the CDC. A lower $f_{\text{CLK}}$ can be selected for applications with relaxed resolution requirements, thereby reducing power consumption. Fig. 9(a) illustrates the CDC output count (CNT) for input currents ranging from 1 *μ*A to 8 *μ*A. At the target frequency of 320 MHz, chosen as discussed in Section III to meet the specified LR error, the CDC achieves a maximum resolution of 150 pA per count. The measurable photocurrent range of the CDC can be tuned by adjusting $I_{\text{DAC}}$, which is controlled through the bias voltage $V_{\text{BIAS}1}$ as shown in Fig. 7. Increasing $I_{\text{DAC}}$ extends the input range, though at the expense of higher power consumption and reduced resolution. Fig. 9(b) illustrates the variation in input current range with different $V_{\text{BIAS}1}$ settings. At $V_{\text{BIAS}1}$, the system achieves an input range of 30 *μ*A, limited by the test setup.

**FIGURE 10.**
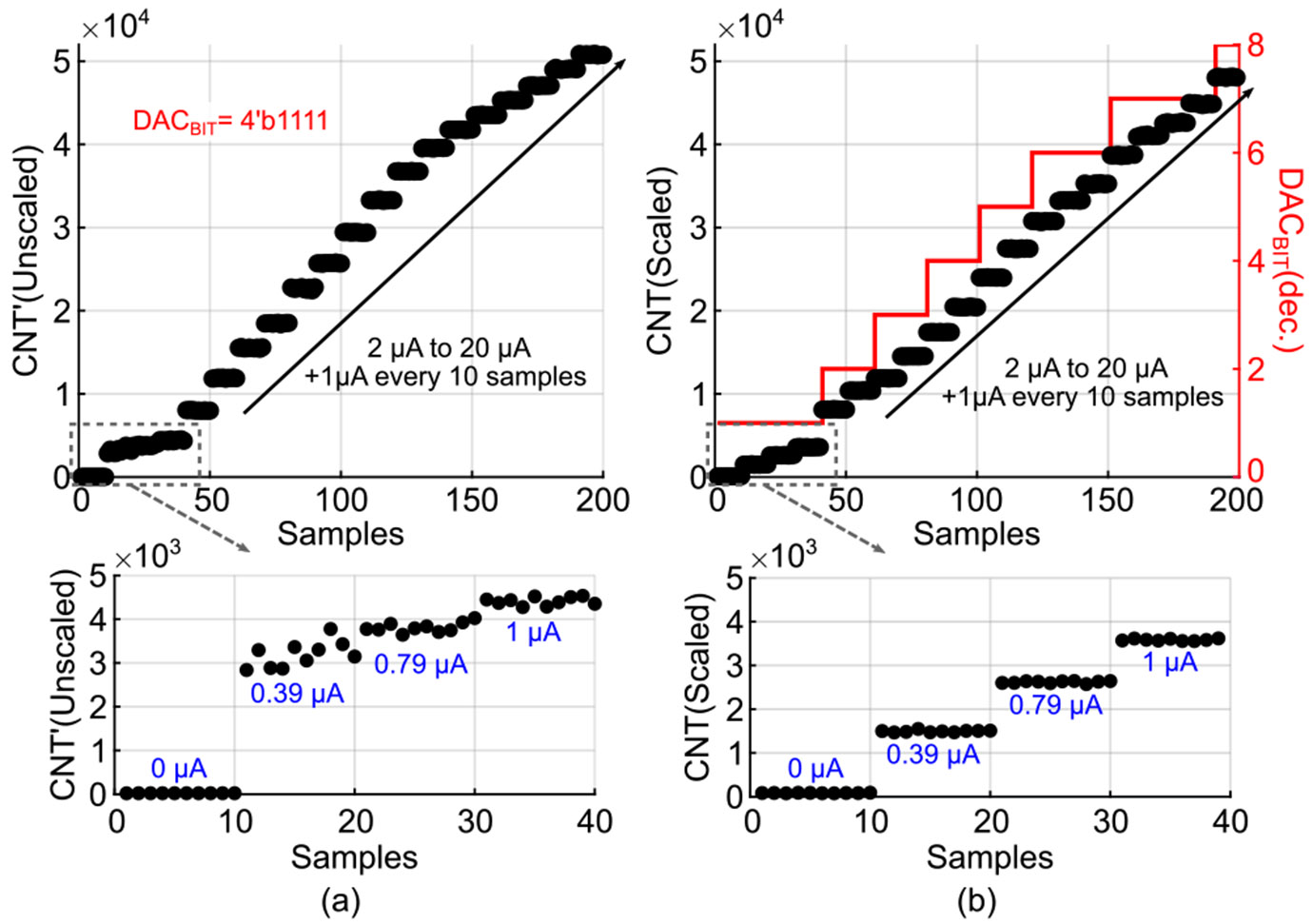
Output of the CDC to a current sweep (a) without and (b) with I-DAC current scaling.

To evaluate the effectiveness of I-DAC current scaling, we repeated the current sweep test with scaling enabled. For this experiment, $f_{\text{CLK}}$ and $V_{\text{BIAS}1}$ were set to 320 MHz and 1.2 V, respectively. Fig. 10(a) shows the CDC output (CNT’) without scaling, where accuracy degrades for input currents below 1 *μ*A. Applying I-DAC scaling improves accuracy at low current levels, as illustrated in Fig. 10(b), thereby enhancing sensitivity. Increased sensitivity enables operation at excitation levels as low as 45 *μ*W from the LED driver, since smaller photocurrents can be reliably detected, thereby reducing overall power consumption. With scaling, lower ${\text{DAC}}_{\text{BIT}}$ values are used for small currents and higher ${\text{DAC}}_{\text{BIT}}$ values for large currents, improving sensitivity without sacrificing the maximum measurable input current. This approach effectively extends the DR, which was measured as 55.2 dB, determined from the ratio between the counts corresponding to the minimum and maximum detectable input currents shown in Fig. 10(b).

**FIGURE 11.**
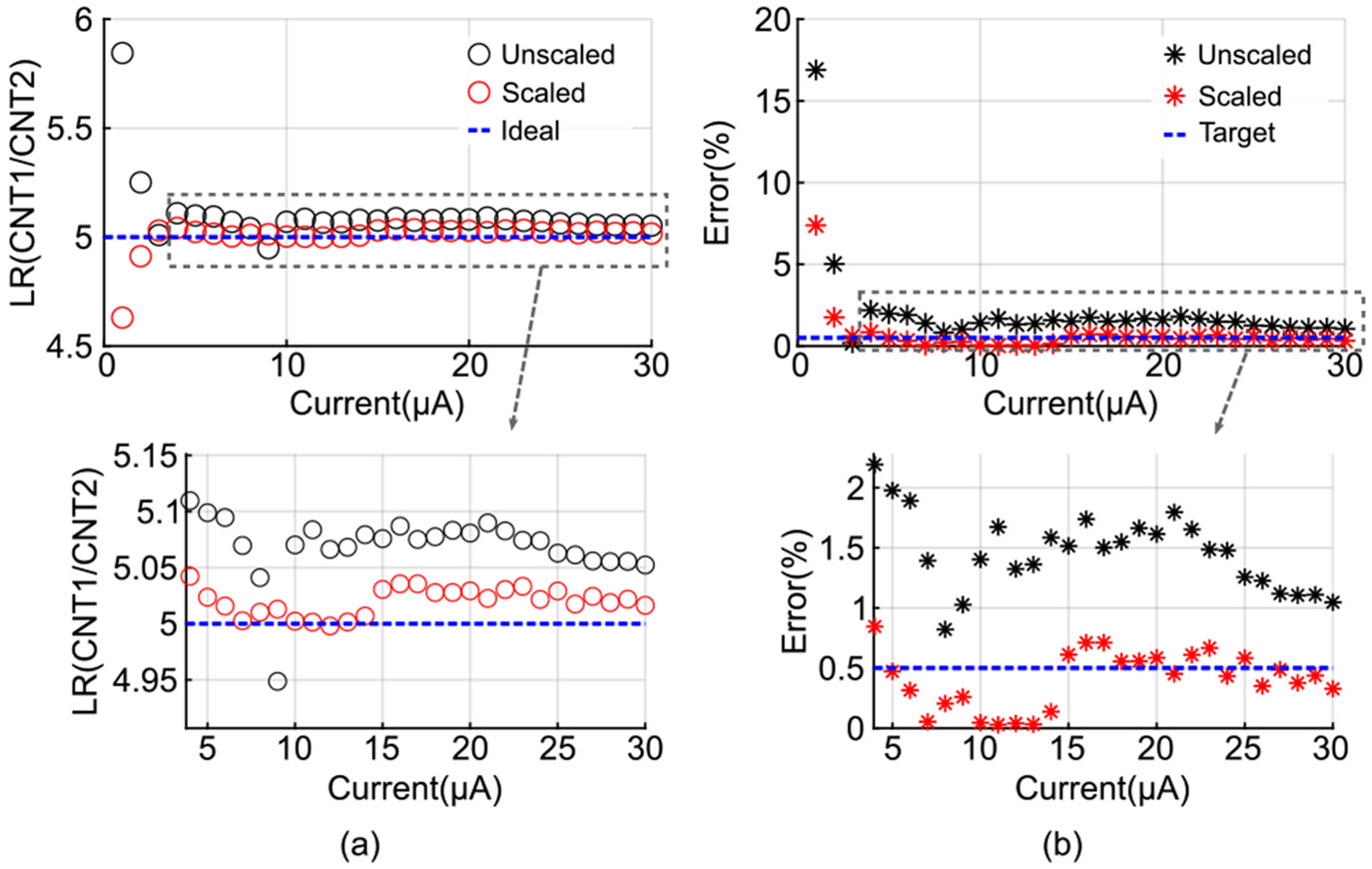
Comparison of the CDC’s performance with and without I-DAC current scaling: (a) LR and (b) error in LR.

We conducted an error analysis to verify compliance with the target LR error of 0.5%. For this evaluation, $V_{\text{BIAS}1}$ was set to 1.15 V to span the full measurable input current range, while $f_{\text{CLK}}$ was kept at 320 MHz. The ratio of current injection durations for CNT1 and CNT2 was fixed at 5, corresponding to an LR of 5, chosen because typical LR values in PCO_2_ monitoring scenarios range from 5 to 10 [9], [12]. Fig. 11(a) presents the measured LR with and without I-DAC current scaling. As shown in Fig. 11(b), applying scaling reduces LR error, keeping it near or below the 0.5% target for most cases. At lower currents, the error increases as expected due to the conversion error and comparator offset becoming more pronounced. The average LR error across all measurements is 0.69%, which improves to 0.46% when currents below 2 *μ*A are excluded.

### B. PCO_2_ SWEEP WITH VARYING EXCITATION

**FIGURE 12.**
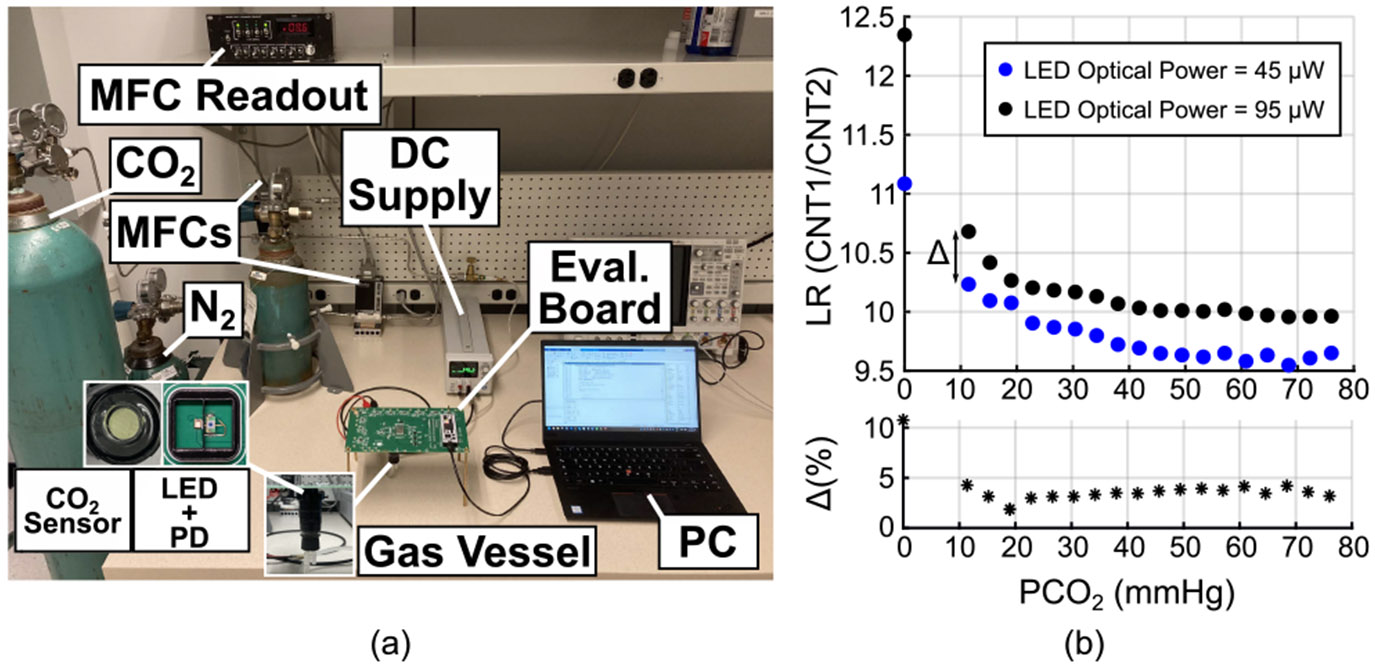
PCO_2_ sweep (a) benchtop setup and (b) results.

The monitoring performance was assessed in a controlled gas vessel with adjustable PCO_2_ levels using the setup shown in Fig. 12(a). Mass flow controllers (MFCs) regulated the CO_2_ and nitrogen (N_2_) flow rates to achieve the desired PCO_2_ inside the vessel containing the sensing film. The precise adjustment of the MFCs is assumed to provide the ground truth for the gas bench experiments. The PCO_2_ was incrementally varied from 0 to 76 mmHg in 3.8 mmHg steps. We should note that the 3.8 mmHg step size reflects the limitation of the MFCs, not an intrinsic limitation of the circuit. To evaluate the robustness of the ratiometric t-DLR approach under different excitation conditions, the LED optical power was set to two levels, such as 45 and 95 *μ*W.

The experimental results are presented in Fig. 12(b). LR measurements at both excitation powers exhibited only ~4% variation across the entire PCO_2_ range, confirming the method’s resilience to even large changes in excitation intensity. While healthy human PtcCO_2_ values typically fall within 35–45 mmHg, they can deviate significantly in individuals with respiratory disorders [32]. As shown in Fig. 12(b), LR remains responsive throughout the full 0–76 mmHg range, detecting 3.8 mmHg increments.

### C. HUMAN SUBJECT EXPERIMENT

**FIGURE 13.**
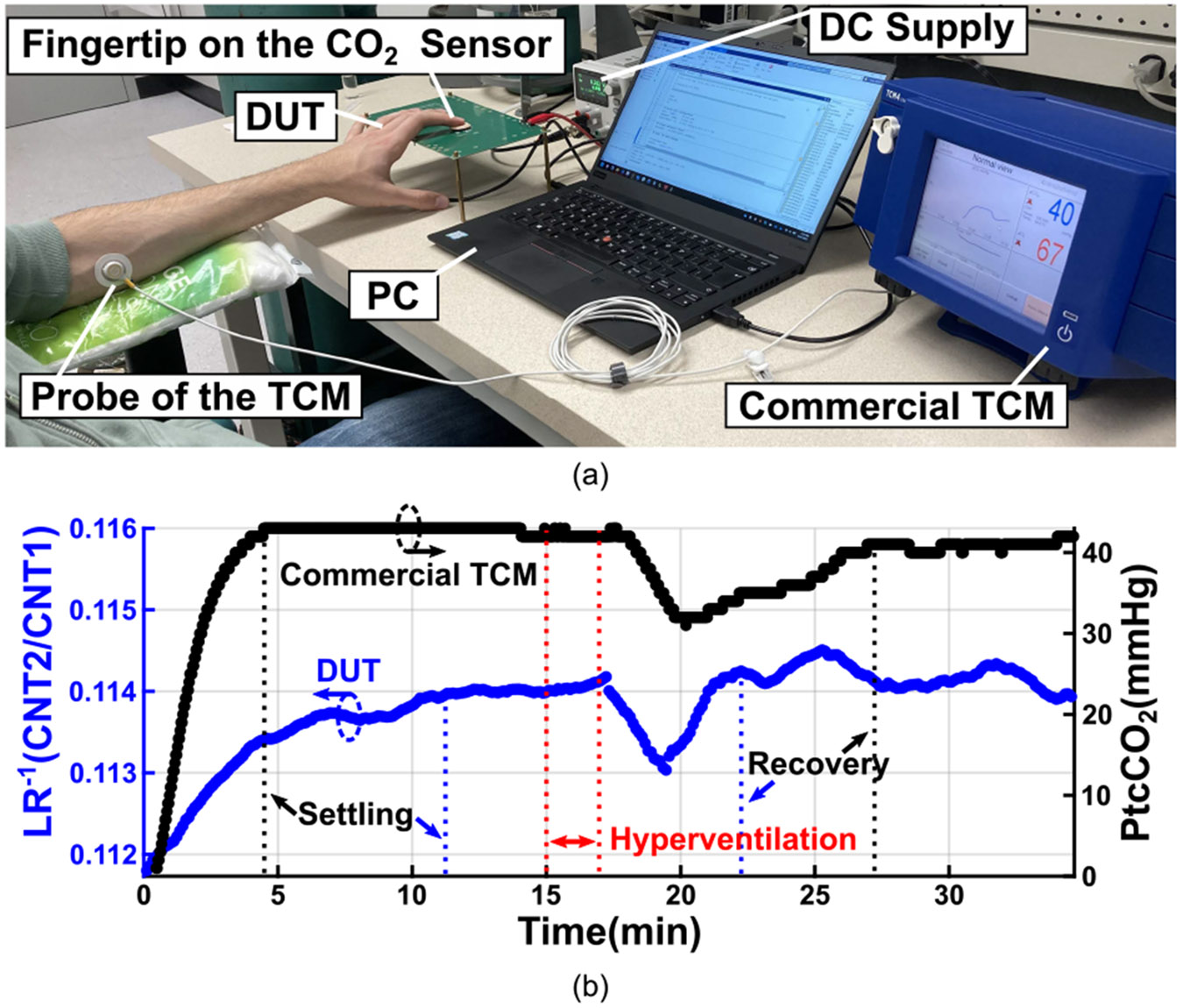
Human subject test (a) setup and (b) results.

We conducted a human subject experiment to evaluate the performance of our system in a real-life PtcCO_2_ monitoring scenario. The Institutional Review Board at Worcester Polytechnic Institute (WPI–IRB) reviewed and approved the experimental protocol (IRB-23-0580), and the subject’s informed consent was obtained prior to the experiment. The experimental setup is illustrated in Fig. 13(a). In addition to our prototype device under test (DUT), we employed a commercial transcutaneous monitor (TCM CombiM, Radiometer Medical ApS) to benchmark our system against an established solution, different from the human subject test reported in [9]. The TCM probe was attached to the subject’s forearm, while the subject’s fingertip was placed on the CO_2_ sensor of our prototype, enabling simultaneous measurements. A MATLAB script controlled the DUT to acquire data at 6-s intervals and to apply a 2-min moving average for smoothing.

Both the TCM and DUT were powered on and allowed to stabilize. As opposed to the TCM, which heats the skin to 44°C to accelerate CO_2_ diffusion through the skin, our prototype operates without skin heating. During the experiment, the TCM consumed an average of 326.4 mW for heating, and achieved stabilization in around 5 min, whereas our system achieved stabilization within about 10 min without additional power for heating. Following stabilization, the subject performed a 2-min hyperventilation exercise to reduce arterial CO_2_ levels, which subsequently lowered PtcCO_2_. As shown in Fig. 13(b), both devices responded to the hyperventilation a few minutes after its onset. The TCM demonstrated a slower recovery, whereas the DUT responded more quickly. Accurate conversion of sensor readings to PtcCO_2_ values requires robust algorithms that account for subject variability, sensor characteristics, temperature, humidity, and other environmental factors. In this work, we report the trend in LR captured by our prototype rather than precise PtcCO_2_ values.

### D. COMPARISON WITH THE STATE-OF-THE-ART

Table 1 provides a comparison between this study and other respiratory sensors, including PCO_2_, partial pressure of oxygen (PO_2_), and photoplethysmography (PPG) sensors. The PCO_2_ sensors from [12] and [29] are discrete implementations, marking our work as the first IC implementation for a PCO_2_ sensor. In addition, our approach is the first IC implementation utilizing the ratiometric, t-DLR method. The PPG sensor presented in [21] employs a ΔΣ-based direct light-to-digital converter (LDC) similar to the CDC architecture proposed in this work. However, it is constrained by a limited input range of 10 *μ*A due its current-self integration scheme, a limitation addressed in our design through a 4b I-DAC with current scaling, achieving a 30 *μ*A input range. Lin et al. [31] reported a noise-shaping (NS) slope LDC that achieves a 134-dB DR while operating at 333 *μ*W with an 1% duty cycle. With the same duty cycling rate, we achieve a competitive power consumption of 88 *μ*W, demonstrating the practicality of creating an energy-efficient wearable using our proposed design. While both [30] and [19] offer a wider sensing range, it is important to consider that the partial pressure of transcutaneous O_2_ (PtcO_2_) range in humans, which is 50–150 mmHg, exceeds that of PtcCO_2_, approximately 35–45 mmHg in healthy individuals. Consequently, PO_1_ sensors need to provide a broader sensing range than PCO_2_ sensors.

## V. CONCLUSION

This work presents the first IC implementation of the ratiometric t-DLR technique using a direct CDC architecture tailored for energy-efficient wearable sensing. The proposed design combines aggressive duty cycling, low LED drive currents, and a compact CDC-based readout to achieve a resolution of 0.15 nA/cnt over a 30 *μ*A input range while consuming only 88 *μ*W. The target LR error of 0.5% is maintained across a wide input current range through an I-DAC current-scaling method. Human subject testing demonstrates performance comparable to a commercial device without requiring a skin heater, significantly reducing power consumption and improving wearability. This work establishes a pathway toward practical, low-power, and miniaturized transcutaneous CO_2_ monitoring for next-generation remote healthcare applications.

## Figures and Tables

**TABLE 1.** Comparison table.

Parameters	[12] 2023	[29] 2021	[30] 2020	[31] 2021	[21] 2017	[19] 2024	This Work
Publisher	TBioCAS	Sensors	ISSCC	ISSCC	ESSCIRC	JSSC	OJ-SSCS
	**Sensor**						
Sensor Type	PCO_2_	PCO_2_	PO_2_	PPG	PPG	PO_2_	PCO_2_
LED (λ)^*a*^	470 nm	IR	460 nm	IR&Red	NR	450 nm	470 nm
LED Current	50-100 mA	NR	25 *μ*A	NR	NR	100 mA	8.3-18 mA
PD (λ)^*a*^	505/600 nm	IR	618 nm	NR	NR	650 nm	505/600 nm
	**Hardware**						
Technology	PCB	PCB	65 nm	180 nm	180 nm	180 nm	180 nm
Chip Area	NA	NA	3.84 mm^2^	8.37 mm^2^	1.79 mm^2^	1.7 mm^2^	5 mm^2^
Readout Technique	Lum. Ratio	IR	Phase Luminometery	Intensity	Intensity	Lum. Lifetime	Lum. Ratio
Fron-End Architecture	TIA	NR	TCA + TDC	LDC (NS-Slope)	LDC (lb ΔΣ)	NUS (C-TIA)	CDC
Supply Voltage(V)	5	3.3	1.2	1.2/3.3	NR	1.8	1.8
Power Comp.(*μ*W)	30100	3400	140	28^*e*^	13~14.4	835	88
Duty Cycle(%)	0.3	NR	NR	1	10	NR	1
Sampling Rate(SPS)	NR	NR	360	NR	160 k	5 M	320 M
Input Range(*μ*A)	NR	NR	NR	200	10	1.5	30
Dynamic Range(dB)	NR	NR	NR	134	NR	NR	55.2
Resolution(nA/cnt)	NA	NA	NA	NR	NR	NA	0.15^*b*^
	**Application**						
Sensing Range(mmHg)	0-76	0-38	3.8-150.5^*c*^	NA	NA	0-240^*c*^	0-76
Human/Animal Testing	Yes	Yes	No	Yes	Yes	Yes	Yes

PCB: Printed Circuit Board, NA: Not Applicable, NR: Not Reported, TCA: Transcapacitance Amplifier, TDC: Time-to-Digital Converter, LDC: Light-to-Digital Converter, NUS: Nonuniform Sampler, a: Peak wavelength, b: Minimum tested, adjustable, c:The range of PtcO_2_ in humans is (50-150 mmHg) is greater than that of PtcCO_2_, d: at 3.8 mmHg, e: AFE power only.

## References

[1] Gattinoni L, Pesenti A, and Matthay M, “Understanding blood gas analysis,” Intensive Care Med, vol. 44, no. 1, pp. 91–93, Jan. 2018. [Online]. Available: 10.1007/s00134-017-4824-y 28497267

[2] Liu Y, Carlson SA, Watson KB, Xu F, and Greenlund KJ, “Trends in the prevalence of chronic obstructive pulmonary disease among adults aged ≥18 years—United States, 2011–2021,” MMWR. Morbidity Mortality Weekly Rep, vol. 72, no. 46, pp. 1250–1256, Nov. 2023. [Online]. Available: http://www.cdc.gov/mmwr/volumes/72/wr/mm7246a1.htm?s_cid=mm7246a1_w 10.15585/mmwr.mm7246a1 PMC10684355 37971940

[3] Linz D, Homberg M, van der Velden RMJ, Bouman E, Buhre W, and Simons SO, “Sleep apnea, obesity and COPD depress respiration during catheter ablation procedures: Implications for transcutaneous carbon dioxide monitoring,” Int. J. Cardiol, vol. 327, pp. 102–104, Mar. 2021. [Online]. Available: https://www.sciencedirect.com/science/article/pii/S0167527320340730 33152419 10.1016/j.ijcard.2020.10.080

[4] Tannoury JE, Sauthier M, Jouvet P, and Noumeir R, “Arterial partial pressures of carbon dioxide estimation using non-invasive parameters in mechanically ventilated children,” IEEE Trans. Biomed. Eng, vol. 68, no. 1, pp. 161–169, Jan. 2021. 32746023 10.1109/TBME.2020.3001441

[5] Tufan TB, Rhein L, and Guler U, “Implementation techniques for transcutaneous carbon dioxide monitoring: Approaches for wearable smart health applications,” IEEE Trans. Biomed. Eng, vol. 71, no. 3, pp. 929–943, Mar. 2024. [Online]. Available: https://ieeexplore.ieee.org/abstract/document/10274108 37812542 10.1109/TBME.2023.3322871

[6] Huttmann SE, Windisch W, and Storre JH, “Techniques for the measurement and monitoring of carbon dioxide in the blood,” Ann. Amer. Thoracic Soc, vol. 11, no. 4, pp. 645–652, Apr. 2014. [Online]. Available: https://www.atsjournals.org/doi/10.1513/AnnalsATS.201311-387FR 10.1513/AnnalsATS.201311-387FR 24701974

[7] Dicembrino M, Barbieri IA, Pereyra C, and Leske V, “End-tidal CO2 and transcutaneous CO2: Are we ready to replace arterial CO2 in awake children?” Pediat. Pulmonol, vol. 56, no. 2, pp. 486–494, 2021. [Online]. Available: http://onlinelibrary.wiley.com/doi/abs/10.1002/ppul.25217 10.1002/ppul.25217 33382537

[8] Sankaran D, Zeinali L, Iqbal S, Chandrasekharan P, and Lakshminrusimha S, “Non-invasive carbon dioxide monitoring in neonates: Methods, benefits, and pitfalls,” J. Perinatol, vol. 41, pp. 2580–2589, Jun. 2021. 34148068 10.1038/s41372-021-01134-2 PMC8214374

[9] Tufan TB, Larkin B, McNeill J, and Guler U, “Luminescence-based transcutaneous carbon dioxide sensor IC using dual-lifetime referencing,” in Proc. IEEE 51st Eur. Solid State Electron. Res. Conf. (ESSERC), Sep. 2025, pp. 1–4.

[10] Klimant I, Huber C, Liebsch G, Neurauter G, Stangelmayer A, and Wolfbeis OS, “Dual lifetime referencing (DLR)—A new scheme for converting fluorescence intensity into a frequency-domain or time-domain information,” in New Trends in Fluorescence Spectroscopy: Applications to Chemical and Life Sciences (Springer Series on Fluorescence), Valeur B and Brochon J-C, Eds. Heidelberg, Germany: Springer, 2001, pp. 257–274. [Online]. Available: 10.1007/978-3-642-56853-4_13

[11] Lakowicz JR, Principles of Fluorescence Spectroscopy, 3rd ed. Berlin, Germany: Springer, 2006.

[12] Tufan TB and Guler U, “A transcutaneous carbon dioxide monitor based on time-domain dual lifetime referencing,” IEEE Trans. Biomed. Circuits Syst, vol. 17, no. 4, pp. 795–807, Aug. 2023. [Online]. Available: https://ieeexplore.ieee.org/document/10128703 37195846 10.1109/TBCAS.2023.3277398

[13] Tufan TB and Guler U, “A miniaturized transcutaneous carbon dioxide monitor based on dual lifetime referencing,” in Proc. IEEE Biomed. Circuits Syst. Conf. (BioCAS), Oct. 2022, pp. 144–148. 10.1109/TBCAS.2023.3277398 37195846

[14] Sant L, Fant A, Stojanović S, Fabbro S, and Ceballos JL, “A 13.2 b optical proximity sensor system with 130 klx ambient light rejection capable of heart rate and blood oximetry monitoring,” IEEE J. Solid-State Circuits, vol. 51, no. 7, pp. 1674–1683, Jul. 2016. [Online]. Available: https://ieeexplore.ieee.org/document/7488199

[15] Sharma A , “A sub-60-*μ*A multimodal smart biosensing SoC with >80-dB SNR, 35-*μ*A photoplethysmography signal chain,” IEEE J. Solid-State Circuits, vol. 52, no. 4, pp. 1021–1033, Apr. 2017. [Online]. Available: https://ieeexplore.ieee.org/document/7820055

[16] Schönle P, Glaser F, Burger T, Rovere G, Benini L, and Huang Q, “A multi-sensor and parallel processing SoC for miniaturized medical instrumentation,” IEEE J. Solid-State Circuits, vol. 53, no. 7, pp. 2076–2087, Jul. 2018. [Online]. Available: https://ieeexplore.ieee.org/document/8331275

[17] Ying D and Hall DA, “Current sensing front-ends: A review and design guidance,” IEEE Sensors J, vol. 21, no. 20, pp. 22329–22346, Oct. 2021.

[18] Rabbani R , “17.3 a fully wireless, miniaturized, multicolor fluorescence image sensor implant for real-time monitoring in cancer therapy,” in Proc. IEEE Int. Solid-State Circuits Conf. (ISSCC), vol. 67, Feb. 2024, pp. 318–320. [Online]. Available: https://ieeexplore.ieee.org/document/10454380 10.1109/jssc.2024.3435736 PMC12311967 40756458

[19] Costanzo I, Sen D, McNeill J, and Guler U, “A nonuniform sampling lifetime estimation technique for luminescent oxygen measurements for biomedical applications,” IEEE J. Solid-State Circuits, vol. 60, no. 8, pp. 2905–2919, Aug. 2025. [Online]. Available: https://ieeexplore.ieee.org/document/10811876 40756063 10.1109/jssc.2024.3512472 PMC12313304

[20] Ferrari G, Gozzini F, Molari A, and Sampietro M, “Transimpedance amplifier for high sensitivity current measurements on nanodevices,” IEEE J. Solid-State Circuits, vol. 44, no. 5, pp. 1609–1616, May 2009. [Online]. Available: https://ieeexplore.ieee.org/document/4907328

[21] Kim H-G and Jee D-W, “A <25 *μ*W CMOS monolithic photoplethysmographic sensor with distributed 1b delta-sigma light-to-digital convertor,” in Proc. 43rd IEEE Eur. Solid State Circuits Conf., Sep. 2017, pp. 55–58.

[22] “S13773 Si PIN Photodiode,” Data Sheet, Hamamatsu Photonics, Shizuoka, Japan, 2019. [Online]. Available: https://www.hamamatsu.com/jp/en/product/optical-sensors/distance-position-sensor/lidar-sensor/si-photodiode-for-lidar/S13773.html

[23] Kahraman B , “Power and accuracy optimization for luminescent transcutaneous oxygen measurements,” in Proc. IEEE Int. Symp. Circuits Syst. (ISCAS), May 2022, pp. 1615–1619.

[24] Technical Manual for Sentec Digital Monitor, Sentec AG, Therwil, Switzerland, 2021. [Online]. Available: https://www.sentec.com/fileadmin/documents/Labeling/Technical_Manuals/HB-005752-t-SDM_Technical_Manual.pdf

[25] TCM TOSCA/CombiM Operator’s Manual, Radiometer Medical ApS, Bronshoj, Denmark, 2024.

[26] Ge X , “Development and characterization of a point-of care rate-based transcutaneous respiratory status monitor,” Med. Eng. Phys, vol. 56, pp. 36–41, Jun. 2018. 29628217 10.1016/j.medengphy.2018.03.009 PMC5932206

[27] Cutaneous Carbon Dioxide (PcCO2) and Oxygen (PcO2) Monitors—Class II Special Controls Guidance Document for Industry and FDA, U.S. Dept. Health Human Services, Food Drug Admin., Silver Spring, MD, USA, Dec. 2002.

[28] Razavi B, “The design of a comparator [the analog mind],” IEEE Solid-State Circuits Mag, vol. 12, no. 4, pp. 8–14, Nov. 2020. [Online]. Available: https://ieeexplore.ieee.org/document/9265306

[29] Tipparaju VV, Mora SJ, Yu J, Tsow F, and Xian X, “Wearable transcutaneous CO2 monitor based on miniaturized nondispersive infrared sensor,” IEEE Sensors J, vol. 21, no. 15, pp. 17327–17334, Aug. 2021. 10.1109/jsen.2021.3081696 PMC8570579 34744520

[30] Sonmezoglu S and Maharbiz MM, “34.4 a 4.5mm^3^ deep-tissue ultrasonic implantable luminescence oxygen sensor,” in Proc. IEEE Int. Solid-State Circuits Conf. (ISSCC), San Francisco, CA, USA, Feb. 2020, pp. 454–456. [Online]. Available: https://ieeexplore.ieee.org/document/9062946/

[31] Lin Q , “28.3 a 28*μ*W 134dB DR 2nd-order noise-shaping slope light-to-digital converter for chest PPG monitoring,” in Proc. IEEE Int. Solid-State Circuits Conf. (ISSCC), vol. 64, Feb. 2021, pp. 390–392.

[32] Storre JH, Magnet FS, Dreher M, and Windisch W, “Transcutaneous monitoring as a replacement for arterial PCO(2) monitoring during nocturnal non-invasive ventilation,” Respir. Med, vol. 105, no. 1, pp. 143–150, Jan. 2011. 21030230 10.1016/j.rmed.2010.10.007

